# Sewing Concrete Device—Combining In-Line Rheology Control and Reinforcement System for 3D Concrete Printing

**DOI:** 10.3390/ma16145110

**Published:** 2023-07-20

**Authors:** Yohan Jacquet, Arnaud Perrot

**Affiliations:** IRDL, UMR CNRS 6027, Université de Bretagne Sud, 56100 Lorient, France

**Keywords:** 3D concrete printing, reinforcement, yield stress, sewing device, in-line quality control

## Abstract

Of the digital concrete-additive-manufacturing techniques, extrusion-based systems are probably the most widespread and studied. Despite the significant potential offered by 3D printing, several challenges must still be overcome. For instance, although several solutions have already been explored, the automated reinforcement of the layer-wise printed structures represents a challenge. The inline quality control of the fresh-state properties of 3D-printed materials is also an open question that needs to be addressed to find an efficient shared practice. This study proposes a new device designed to simultaneously reinforce 3D-printed structures along and through the layers and to be used as an inline quality-control device. This device consists in a sewing system, which is composed of a rotating system, and a hollow needle, which drives a reinforcing cable or yarn and can be used to inject cement grout to fill holes and improve bonding with reinforcement. The rotation is induced by a stepper motor, which measures the torque that is required to make the needle penetrate. This measurement can be used as a quality-control index to ensure material homogeneity. This paper aims to present an original reinforcement system that can be fully automated and simultaneously create reinforcement patterns in different directions of the printed structure while controlling the material’s fresh properties.

## 1. Introduction

Three-dimensional (3D) concrete printing is a rapidly developing technique that has attracted significant attention from researchers in recent years. Nevertheless, several questions concerning this digital additive-manufacturing process remain unanswered, such as the structural design or the development of quality-control methods. As a result, the structural design of 3D-printed concrete is still challenging because of the lack of design codes, a consensus about the safety coefficient, and a well-documented reinforcement method.

Taking advantage of the freedom of shape offered by 3D concrete printing to design and optimize structures subject only to compression, as in the case of double-curvature walls, for example, could be an ideal application for 3DCP [1,2,3,4]. Nevertheless, in most construction projects, loadings induce shear and tensile stresses, which need to be taken into account in structural designs, making the reinforcement of printed concrete structures mandatory to overcome concrete’s weakness under tension [5,6,7]. Large-scale structures, such as bridges, walls, and beams need to be reinforced to meet mechanical requirements [8].

Classifications of reinforcing methods have recently been proposed by different research groups [9,10]. It is suggested that reinforcing methods can be differentiated according to the timepoint at which the reinforcement is placed during the printing process and the nature of the reinforcing elements.

In this regard, three categories of reinforcing method can be distinguished: the first consists in entraining the reinforcements in the concrete flow simultaneously to the printing [11] (embedded reinforcements, like fibers and cables can be included in this classification); the second consists in placing the reinforcement before the concrete printing [12,13]; and the third and last method aims to reinforce already deposited layers of concrete by inserting or screwing reinforcements (nails, staples, screws, rebars, etc.) in different directions [14,15,16,17,18]. However, this last reinforcing method needs the implementation of multiple robots or tools (for instance one robot for concrete printing and one robot for reinforcement) and leads to complexity in robotic control and programming [19,20].

Several researchers have already explored these different methods of reinforcement, from pre-stressed post-assembled concrete elements [21] to non-automated reinforced 3D-printed concrete [5]. Of the multiple currently explored reinforcement techniques, the most promising, especially considering the potential of automation, consists in embedding reinforcements directly in printed cementitious materials. In this strategy, a large panel of reinforcements can be used, from fibers and rods [22,23,24,25] to continuous cables [26], yarns, or woven textiles [27,28]. The use of fibers as a method of reinforcement for concrete structures is a common and fully documented practice [29], but it requires a control of the fibers’ orientation during extrusion and deposition [30,31]. Therefore, alternative methods that consist in the use of continuous reinforcements, such as unrolled filaments or yarns, have been developed.

Different materials can be used to reinforce concrete, such as steel [23], polymers [32], basalt [33,34], glass [35,36], carbon [37], or even natural fibers [38], and they have already been tested, often with the same observation that they increase the strength and ductility of concrete structures. It is also well known that the bond strength between concrete and fibers is a key parameter in the reinforcement efficiency. It is worth noting that the same phenomenon is encountered between concrete and inserted steel bars [17]. A previously explored approach to increase this adhesion was the impregnation of these fibers with cement grout before processing [39,40]. Alternatively, the grouting of cement paste around the reinforcement can be an efficient solution [17].

Another question to which an answer is still pending is the organization of the quality control of the process that is needed to check the homogeneity of t concrete and the fresh properties of materials before and after printing [41,42,43]. Different offline or inline testing methods can be used to control the initial consistency of the printable mortar, like the slump test in the case of conventional cast concrete [44]. For example, slug tests, which consist in measuring the mass of the extrudates that breaks under its own weight at a vertically oriented nozzle exit, is an easily implementable and promising method to control the initial shear-yield stress of materials immediately before printing [45,46]. Nevertheless, other types of test need to be developed in order to control materials after their deposition and the structural build-up of materials to prevent the collapse of printed structures due to buckling or to plastic failure in the base layer [47,48,49].

In this paper, a new device is presented to simultaneously control deposited materials’ consistency and reinforce printed materials. The reinforcement strategy consists in sewing the deposited layers with a continuous yarn to create a reinforcing pattern. The interface between the yarn and the cementitious material can be improved by the injection of cement grout through a hollow sewing needle. Moreover, the developed device can be used for the in-line control of the fresh-state properties of deposited mortar by measuring the load required to ensure the needle penetrates the layers.

This study explores the ability of in-line rheological characterization, which is allowed by sewing concrete devices, to compute shear-yield stress, and provides some preliminary results regarding the reinforcement efficiency of the proposed method.

A three-point flexural test was performed on printed beams to assess the effect of the reinforcement on the mechanical behavior. A comparison between different reinforcement densities and patterns, orientations of yarns, and bonds between materials and yarns was conducted in order to provide to the reader with a glimpse of the wide range of applications offered by the sewing concrete device—SCD. Additionally, the ability of this device to measure the yield stress of the deposited material is also examined.

## 2. Materials, Devices and Methods

### 2.1. Materials

The following mix design was used: cement CEM I (22% by dry volume), sand (78% by dry volume), tap water at a water-to-cement ratio W/C = 0.55, and a high-range water-reducing admixture at a dosage of 1.5% of the cement content. Finally, to ensure rheological requirements of printable material, a viscosity-modifying admixture in powder form, based on hydroxypropylmetylcellulose (15,000 mPa·s of grade), was added to the mixture following a dosage of 0.675% of the water mass. 

The yarn selected to conduct these experiments was a polyethylene-based braided continuous yarn 0.35 mm in diameter, displaying a tensile strength of 2.65 ± 0.1 GPa.

In some of the prints, a cement paste was grouted to improve the interface between the yarn and the printed mortar. This cement paste was the same as that used in the mortar composition, with the same admixture contents. The cementitious materials were prepared using a four-step procedure. Dry materials were first mixed using a planetary Hobart mixer for two minutes at low velocity (64 rpm). Next, the water was added slowly, and the material was mixed at low velocity for 1 min. The rotation velocity of the mixer was then increased to its highest level (256 rpm), at which point the material was mixed for 3 min. The mixer was stopped to scrap the bowl and remove material that remained stuck at the wall. The last step consists in a final 2-minute mixing period at high velocity. After preparation, the material was printed for 60 min.

### 2.2. The Sewing Device

The sewing concrete device, SCD, is a technical solution able to simultaneously reinforce concrete during printing and perform in-line quality control of freshly deposited materials.

**Figure 1:**
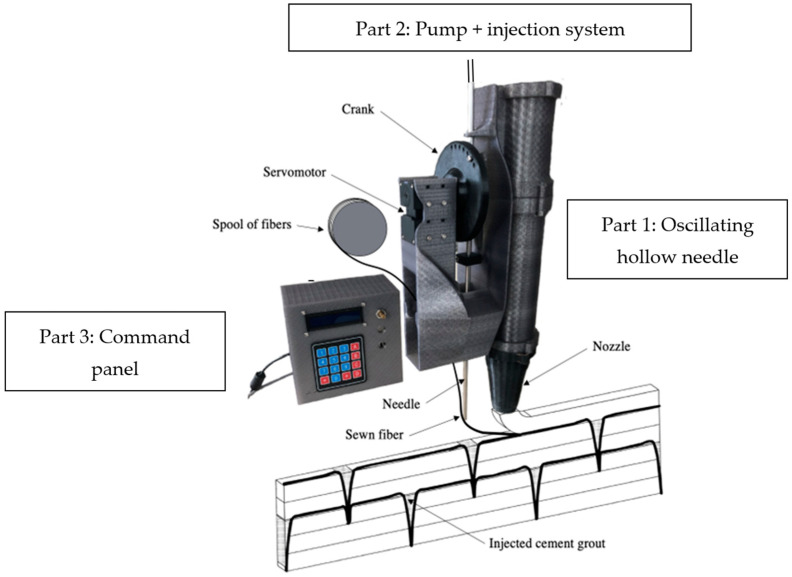
Description of constituent parts and patterns of the sewing device.

**Figure 2:**
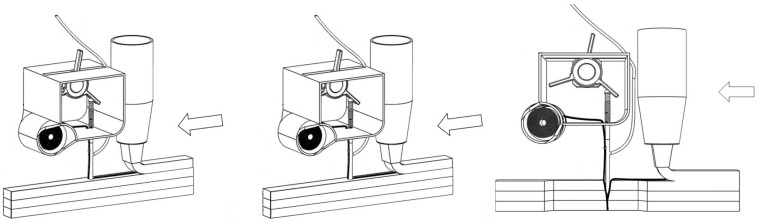
Sewing concrete device equipped with stepper motor and rocker to control needle oscillation along the printing path.

The sewing concrete device is divided into three parts (Figure 1): the first consists in an oscillating hollow needle attached to a slider crank mechanism, which is itself driven by a motor. The second part is the system used to inject cement grout around the yarn to improve the bonding with the cementitious material. It consists in a diaphragm pump, which is attached to the top of the hollow needle by a silicone tube. The last part is a control panel, which allows control over oscillation frequency, penetration depth of the needle (i.e., correlated with the motor angular deflection), and continuous retrieval of the torque data at the level of the motor shaft. Oscillation of the needle can be driven by two methods: linking the stepper motor to a rocker (Figure 2), or to a slider–crank mechanism (Figure 1). Whichever method is used, an elastic spring can be advantageously added to the system to ensure that the needle quickly returns to its initial top position. 

The needle consists in a hollow stainless-steel tube 6 mm in diameter, which is equipped with a 3D-printed concave shape tip. It is necessary to tune the tension in the unwound yarn to avoid loss of contact with the tip of the needle and allow a constant rate of yarn unwinding. The layer-pressing strategy, for example [50], can fulfill this condition, but it requires control over the path and position of the sewing concrete device to ensure that the needle is positioned immediately in front of the nozzle on the same printing path (see Figure 1), and it necessary for the distance between each penetration location to be greater than the distance between the needle and the nozzle. It is therefore better to use another axis of freedom (i.e., the orientation of the nozzle in the direction perpendicular to the deposition plan) to reap the full benefits of this device.

It is important to note that this device offers the possibility of varying the distance between each penetration, the penetration depth, the yarn’s nature, and geometry. Several kinds of continuous fiber or yarn can be used, from synthetic to bio-based, and from non-braided to braided, or even stranded for metal applications. In addition, adhesion between surface of the yarn and the cementitious material can be improved by the cement grouting allowed by the geometry of the hollow needle. This grouting solution can be used to prevent lack of adhesion between the reinforcement and the matrix, which can significantly affect the ability of the matrix to transfer mechanical loads from concrete to the reinforcement and the structural efficiency of the printed elements.

The force required by the motor to move down the needle and insert it in the material can be estimated and, thus, it can be used to indirectly estimate the shear stress induced by the penetration. The servomotor allows measurement of the torque that is needed to make the needle penetrate inside the deposited layers. This torque value is used to compute the penetration stress, which is itself used as a quality-control parameter for the initial material properties.

**Figure 3:**
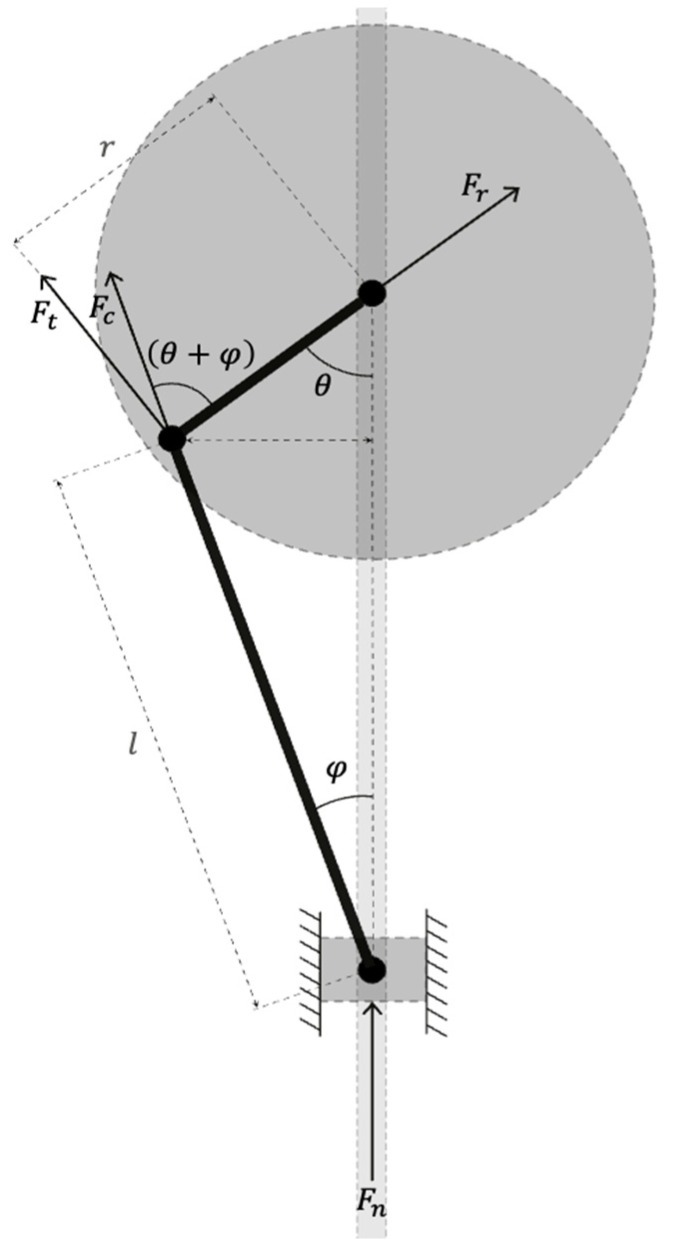
Sewing concrete device: schematic representation of the mechanism.

The sewing device consists in a slider–crank mechanism composed of a slider attached to the needle and a crank linked to the servomotor; a geometrical representation associated with the SCD is proposed in Figure 3. The torque T can be continuously measured due to a torque sensor equipped inside the servomotor. Using the torque T, it is possible to compute the normal force transmitted to the needle and then the shear stress at the interface between the needle and the printed material. Torque data are recorded using U2D2 adapter and the associated software RoboPlus. The pressure exerted on the slider is related to the penetration force and, thus, to friction along the needle when penetrating material: it linearly increases with the penetration depth.

A servomotor, Dynamixel XH540-W150T, with a torque capacity of 7.1 N·m (i.e., the maximum torque that servomotor is able to measure with built-in sensor) was used in this study. This torque capacity determines the maximum number of layers that the needle can penetrate. Considering the capacity of the built-in torque transducer and the common intended use of this device, the experiments presented in this study only considered cases in which needle penetrates through a maximum of four layers.

The penetration system is submitted to two forces: the force exerted by the needle on the slider $F_{n}=F_{c}\cos\varphi$, the force transmitted to the connecting rod $F_{c}$. In addition, it is subjected to two further forces, the tangential $F_{t}=F_{c}\sin\left(\theta +\varphi \right)$ and the radial $F_{r}=F_{c}\cos\left(\theta +\varphi \right)$ ones which are perpendicular to the crank. The rotation is activated by the servomotor shaft with a torque T, which needs to overcome the resisting moment, which is equal to $F_{c}$ multiplied by r, the radius of the crank. The correlation between torque at the servomotor shaft and the penetration force of the needle is given by Equation (1).

(1) T=Fncosϕ sinθ+φ.r=Fn . r . sinθ+sin2θ2 lr2−sin2θ

The penetration of a needle into the fresh printed material creates friction along the cylindrical section of the immersed needle (with an outer diameter equal to D=6 mm). Therefore, the friction force, which is equal to $F_{n}$, progressively increases with the penetration depth $h_{pen}$. Assuming that the flow is static, the normal force transmitted to the needle when penetrating the printed element can be linked to friction shear stress on the material, according to Equation (2).

(2) $$F_{n}=\tau_{y}\;\pi \;D\;h_{pen}$$

Combining Equations (1) and (2), the torque value measured at the level of servomotor shaft can be linked to the friction shear stress $\tau_{y}$ on the material using Equation (3).

(3) τy=Tπ D hpen rsinθ+sin2θ2 lr2−sin2θ

Depending on the time between each deposit of successive layers (i.e., related to the length of printing path of a single layer), the structural build-up of the printed concrete can thus be monitored by measuring the torque needed to penetrate more than one layer. This means that it is possible to study the layered structure with a discretized gradient of friction-shear-stress values according to the time since deposit. This strategy is similar to that developed in the study by Perrot et al. for the prediction of nails’ penetration forces [14].

### 2.3. Printed Elements and Reinforcing Patterns

A WASP 3DMT printer was used to print samples used in this study. It is a delta robot equipped with a feeding system, which consists in an endless screw attached to a 5 L hopper. The printing strategy used a mono-component approach.

In order to study the reinforcing effect of the sewn yarn, rectangular-cross-section beams were printed. Seven 0.5-m-eter-long mortar beams with cross sections measuring 0.1 m × 0.1 m were printed with a layer height of 10 mm (for a nozzle diameter of 0.025 m and an extrusion rate of 0.023 m·s^−1^).

**Figure 4:**
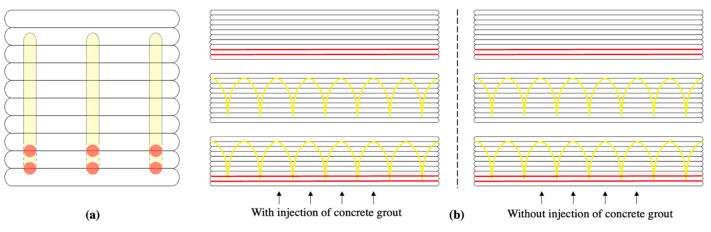
Cross-sectional view (**a**), and side views (**b**) of the different strategies of reinforcement investigated—continuous fiber at the interlayer level (red) and sewn fiber through multiple layers (yellow).

Reinforcement of 3D-printed concrete elements involved placement of yarns along and through the different layers to sustain bending and shear loads, respectively. In this study, two ways of adding yarns to the printed structures were tested. The first solution consisted in depositing the yarn without using the needle to make the yarn penetrate through the layers, and the second consists in sewing the yarn through the layers by using the needle moved by the constant rate rotation of the motor. These two yarn-addition strategies were tested separately and in combination, as shown in Figure 4.

Therefore, a prospective study is reported in this paper. Three different yarn-based reinforcement patterns were tested. The first consisted in depositing yarns horizontally along the upper surfaces of the first and the second printed layers (red lines in Figure 4). The second reinforcement patten consisted in a yarn sewn between the second and the second-to-last layers at a penetration depth of 0.03 m a distance between penetrations of 0.12 m (yellow lines in Figure 4), while the last pattern was based on the combination of the first and second strategies.

For these three strategies, the influence of the bonding between yarns and cementitious matrix was assessed by printing the reinforced beams with and without cement-grout injection through the hollow needle. This grouting was expected to minimize the remaining porosity induced by the needle penetration and to increase the adhesion between yarns and printed mortar at the same time. Finally, by means of comparison, a last sample was printed without any reinforcements to obtain a sample displaying brittle behavior.

### 2.4. Bending Tests and Digital-Image Correlations

The bending loads at failure of the printed beams were measured using a three-point bending device with a distance between supports of 0.44, with a centered vertical load application. This device was mounted on a 50 kN loading machine. The tests were carried out at a constant loading rate of 1 mm/min in order to approximate quasi-static conditions [50].

Preparation of samples consisted in sawing longitudinally printed beams after 28 days of curing (in a controlled environment at 20 °C and with 60% relative humidity) to obtain three slenderer beams (whose cross-sections were 0.03 m wide and 0.1 m high) with only one sewing pattern instead of three (as shown in Figure 4). For each reinforcing pattern and with and without grouting cement paste, only one bending-load value is reported: the average value of the three bending-test results. In addition to the average value, the computed standard deviation is also provided.

Moreover, reference unreinforced samples were printed without any yarn, but with and without grout injection at the layer interface in order to assess the effect of the grout on the unreinforced samples to improve layer-to-layer bonding. Therefore, a total of 8 samples were printed and mechanically tested.

In order to obtain more information on the beams’ bending behavior, digital-mages correlation was used as a non-destructive method to control and evaluate the deformation ability of the reinforced elements. The choice was made to use this system only when cement-grout injection was performed. The digital-image-correlation system was composed of cameras with lenses 50 mm in focal length. Each sample surface was smoothed, painted, and speckled to enhance contrast. The three reinforcement patterns were thus compared with the reference printed sample. Due to digital-image correlation, the mid-span crack opening at the bottom of the beam was thus quantified for each sample and used as a first insight into the reinforcing effect of the sewn yarns in 3D-printed beams.

### 2.5. Shear-Yield-Stress Measurements

To check the validity of the in-line quality-control measurements, a reference rheological test was also performed using a vane 22 mm in diameter and 40 mm in height. Material was placed in a cup 34 mm in diameter (i.e., gap width of 6 mm). The vane-shear test was performed at a constant rotational speed, which was close to that of the needle during penetration (i.e., 1 rpm). This test was performed after 10 min of resting time, which corresponded to the printing time, in order to ensure comparable structural build-up of the material. Computation of ultimate shear stress from torque regarding vane geometry was performed by following Equation (4).

(4) $$\tau_{y}=\frac{T}{2\pi R^{2}\left(h+\frac{R}{3}\right)}$$

The tests were performed in three replicates and the average value was computed.

## 3. Results

### 3.1. Sewn Yarns’ Reinforcing Ability

Table 1 summarizes all the average values of the ultimate bending loads and Table 2 provides the crack opening at mid-span on the bottom of the beam.

The reference sample, printed without any reinforcements, exhibited an ultimate bending load of 0.65 ± 0.04 kN for a computed Von Mises bending strain at a fracture of 0.36% without cement-grout injection, and 0.82 ± 0.06 kN with a strain at fracture near to 0.40% with cement-grout injection. These results show that the injection of grout into the unreinforced samples did not significantly affect their bending behavior.

**Figure 5:**
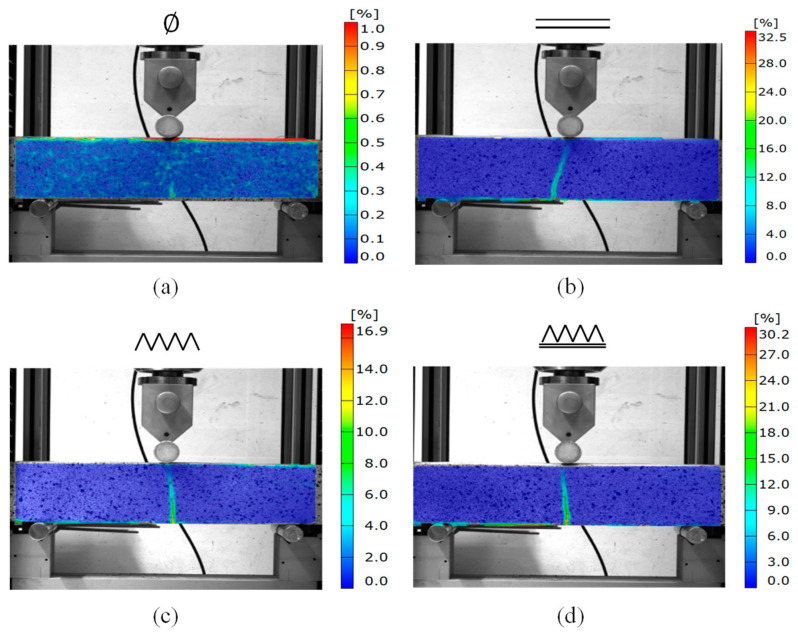
Mises-strain measurements using digital-image correlation on printed samples without reinforcements (**a**), with fiber deposited on the layers (**b**), with only fiber sewn through different layers (**c**), and with both reinforcement strategies (**d**).

When reinforcements were added, the mechanism of failure changed: the sample developed a crack at the bottom without breaking, while it directly broke without reinforcement (fragile behavior). The reference printed beam (Figure 5a) exhibited very brittle behavior, with a critical strain near 0.4%, and did not display any recordable cracks. However, the three others presented a huge displacement at midspan on the bottom stretched line due to the cementitious material cracking. As expected, the first pattern of deposited horizontal yarns (Figure 5b) provided bending resistance to the beam, as shown by the crack opening. The lattice pattern sewn through the layers (Figure 5c) showed a lower crack opening at failure, but the combination of both technics (Figure 5d) induced higher crack opening.

The comparison between the results obtained from the reference sample with those obtained using the longitudinal horizontal reinforcements, with the deposition of continuous yarns on the surfaces of the layers (i.e., the red lines in Figure 4), showed an increase in bending strength with the addition of the yarns. These interlayer reinforcements doubled the ultimate bending load while increasing the crack opening at failure to 8.2 mm. In addition, the improvement in the interface between the yarns and the layers using cement grouting made it possible to increase the load capacity by 120% compared to the samples that only had deposited continuous yarns. This showed that the interface between the yarns and the matrix affected the overall behavior of the printed composite.

It is also worth exploring the effect of the sewing of the fiber through different layers (the yellow lines in Figure 3), with and without cement-paste grouting, on the improvements in the interfacial bonding. Compared to the reference sample, the yarns sewn through six to seven layers allowed an increase in the bending ultimate load of 170% while increasing the crack opening at failure by 13%. Moreover, the cement-grout injection during sewing led to an additional increase in the bending strength, of 125%.

It was also of interest to study the effect of the complex pattern made of the combination of deposited and sewn yarns (red and yellow lines in Figure 4). This strategy made it possible to reach an increase in the ultimate bending load of 260% compared to the reference value, and with a crack opening at failure close to 10.5 mm. In a manner that was similar to the other reinforcing patterns, the cement-paste grouting made it possible to increase this ultimate bending load by more than 120%. This strategy, based on creating a lattice of reinforcing yarns, seems to have exhibited high potential regarding the structural reinforcement of printed elements.

Regarding the tensile strength of the polyethylene-based yarn of 2.65 GPa and its diameter of 0.35 mm, the fiber was always broken during the tests (there was only minor yarn debonding). Moreover, in this study, the mass ratio between the yarns and the printed mortar was lower than 1%, showing the efficiency of the reinforcing solutions at such low contents. This reinforcing ratio can be significantly increased to ensure better mechanical ability. This study provides a first insight into the influence of sewn yarns on the reinforcement of printed structures, and the results obtained are very promising.

### 3.2. In-Line Quality Control Using Rheological Measurements

The penetration occurred inside four layers, the penetration depth was h_pen_ = 40 mm, and the angle θ was equal to 23.6° (see Figure 4). The torque data were recorded continuously every 0.25 s during the printing and sorted to keep only the values higher than 1 mN.m and neglect values recorded outside the penetration step. The mean value and deviation of the penetration forces were computed from seven oscillations of the needle in the four layers of the printed material. For these in-line penetration tests, the measured mean torque value was equal to 46 ± 8 mN·m, which, once computed using Equation (3), gave an ultimate shear stress equal to 957 Pa ± 167 Pa. This protocol can be extended to follow the hardening of concrete through four layers until the material reaches an ultimate shear stress equal to 148 kPa.

It is worth noting that in our study, the mortar mix was not accelerated, and the printing path was short. Therefore, the time taken to print the beam was also short, and the structural build-up was thus neglected. It was therefore assumed that the ultimate shear stress of the mortar could be considered constant through the different layers penetrated by the needle.

Three repetitions of the vane-test measurements showed a mean value of ultimate shear stress equal to 814 Pa ± 71 Pa (using Equation (4)), which was close to that given by the in-line-penetration control. The gap between the in-line measurement and the conventional rheological test was probably related to differences in the strain rate. Since this in-line solution was only intended approximate the rheological values and control the homogeneity of the printed material during the process, this measurement error was considered acceptable.

## 4. Conclusions

This paper aims to highlight the potential of a new device to simultaneously automate the reinforcement of printed elements in all directions during the printing process and perform the in-line quality control of the properties of printed concrete.

This study presents the development of a fully automatable device that was initially used to reinforce printed structures along and through layers using a continuously deposited and sewn yarn (continuous fiber). A demonstration of the technical possibilities was carried out using a common 3D-printed mortar and a polyethylene-based yarn of braided fibers. However, this sewing device can easily be expanded to other printed materials, such as earth-based materials, for instance, and other kinds of fiber, from bio-based fibers (such as flax, hemp, …) to braided-steel cables, in the most demanding structural applications.

Equipped with a printer, this system, called the sewing concrete device, or SCD, makes it possible to efficiently follow a printing path. Moreover, the frequency and amplitude of the needle can easily be managed to adjust the reinforcement density in relation to the intended mechanical loads. Lastly, from a purely mechanical point of view, the injection of cement grout during the deposition and sewing of fiber makes it possible to avoid well-known phenomena in the field related to the weaknesses of the interlayer (often referred to as the “cold joint”) and to the interface between reinforcements and cementitious materials. This grouting makes it possible to significatively increase the cohesion between yarns and extruded materials and it seems, therefore, to enhance the overall mechanical strength of printed samples.

Finally, since this reinforcing system is based on the controlled penetration of a hollow needle through different layers, the rheological properties of printed materials can be determined from data recorded by the electronic parts of the SCD. A slider–crank system is placed on a servomotor to measure the instantaneous torque transmitted when the needle enters a set of printed layers. This in-line rheological measurement paves the way towards fully automated in-line quality-control systems for the 3D printing of construction materials, making it possible to check materials’ fresh properties during the whole process.

As shown in this paper, the most important technical barriers have been overcome. However, further development would consist in the scaling up of the device for large concrete 3D-printed elements and industrial uses.

## Figures and Tables

**Table 1:** Ultimate bending loads of printed reinforced elements using three-point bending test, with and without cement-grout co-extrusion.

Pattern	Cement-Grout Injection	Bending Load, kN	Deviation, kN
∅	No	0.65	±0.04
∅	Yes	0.82	±0.06
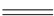	No	1.29	±0.13
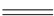	Yes	1.56	±0.07
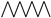	No	1.13	±0.16
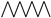	Yes	1.41	±0.09
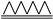	No	1.68	±0.11
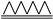	Yes	2.04	±0.09

**Table 2:** Von Mises strains, computed using digital-image correlation, of reinforced elements with cement grouting.

Pattern	Cement-Grout Co-Extrusion	Bending Strength, kN	Crack Opening, mm
∅	Yes	0.65	-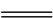	Yes	1.55	8.2
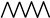	Yes	1.41	6.3
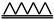	Yes	2.04	10.6

## Data Availability

The data presented in this study are available on request from the corresponding author.

## References

[1] Perrot A., Amziane S. (2019). 3D Printing in Concrete: General Considerations and Technologies. 3D Printing of Concrete: State of the Art and Challenges of the Digital Construction Revolution.

[2] Motamedi M., Oval R., Carneau P., Baverel O. (2020). Supportless 3D Printing of Shells: Adaptation of Ancient Vaulting Techniques to Digital Fabrication. Design Modelling Symposium Berlin.

[3] Buswell R.A., de Silva W.R.L., Jones S.Z., Dirrenberger J. (2018). 3D printing using concrete extrusion: A roadmap for research. Cem. Concr. Res..

[4] Schwartz J. (2018). Graphic statics and their potential for digital design and fabrication with concrete. Cem. Concr. Res..

[5] Asprone D., Menna C., Bos F.P., Salet T.A., Mata-Falcón J., Kaufmann W. (2018). Rethinking reinforcement for digital fabrication with concrete. Cem. Concr. Res..

[6] Bos F.P., Ahmed Z.Y., Wolfs R.J.M., Salet T.A.M., Hordijk D.A., Luković M. (2018). 3D Printing Concrete with Reinforcement. Proceedings of the High Tech Concrete: Where Technology and Engineering Meet, Maastricht, The Netherlands, 12–14 June 2017.

[7] Menna C., Mata-Falcón J., Bos F.P., Vantyghem G., Ferrara L., Asprone D., Salet T., Kaufmann W. (2020). Opportunities and challenges for structural engineering of digitally fabricated concrete. Cem. Concr. Res..

[8] Bos F.P., Menna C., Pradena M., Kreiger E., da Silva W.R.L., Rehman A.U., Weger D., Wolfs R.J.M., Zhang Y., Ferrara L. (2022). The realities of additively manufactured concrete structures in practice. Cem. Concr. Res..

[9] Mechtcherine V., Buswell R., Kloft H., Bos F.P., Hack N., Wolfs R., Sanjayan J., Nematollahi B., Ivaniuk E., Neef T. (2021). Integrating reinforcement in digital fabrication with concrete: A review and classification framework. Cem. Concr. Compos..

[10] Kloft H., Empelmann M., Hack N., Herrmann E., Lowke D. (2020). Reinforcement strategies for 3D-concrete-printing. Civ. Eng. Des..

[11] Mechtcherine V., Grafe J., Nerella V.N., Spaniol E., Hertel M., Füssel U. (2018). 3D-printed steel reinforcement for digital concrete construction–Manufacture, mechanical properties and bond behaviour. Constr. Build. Mater..

[12] Asprone D., Auricchio F., Menna C., Mercuri V. (2018). 3D printing of reinforced concrete elements: Technology and design approach. Constr. Build. Mater..

[13] Hack N., Lauer W.V. (2014). Mesh-Mould: Robotically Fabricated Spatial Meshes as Reinforced Concrete Formwork. Arch. Des..

[14] Perrot A., Jacquet Y., Rangeard D., Courteille E., Sonebi M. (2020). Nailing of Layers: A Promising Way to Reinforce Concrete 3D Printing Structures. Materials.

[15] Hass L., Bos F. (2020). Bending and pull-out tests on a novel screw type reinforcement for extrusion-based 3D printed concrete. Proceedings of the RILEM International Conference on Concrete and Digital Fabrication.

[16] Hass L., Bos F. (2022). Robotically Placed Reinforcement Using the Automated Screwing Device–An Application Perspective for 3D Concrete Printing. Proceedings of the RILEM International Conference on Concrete and Digital Fabrication, Loughborough, UK, 27–29 June 2022.

[17] Freund N., Dressler I., Lowke D. (2020). Studying the bond properties of vertical integrated short reinforcement in the shotcrete 3D printing process. Proceedings of the RILEM International Conference on Concrete and Digital Fabrication.

[18] Cao X., Yu S., Cui H. (2022). Experimental Investigation on Inner-and Inter-Strip Reinforcements for 3D Printed Concrete via Automatic Staple Inserting Technique. Appl. Sci..

[19] Dörfler K., Dielemans G., Lachmayer L., Recker T., Raatz A., Lowke D., Gerke M. (2022). Additive Manufacturing using mobile robots: Opportunities and challenges for building construction. Cem. Concr. Res..

[20] Zhang X., Li M., Lim J.H., Weng Y., Tay Y.W.D., Pham H., Pham Q.-C. (2018). Large-scale 3D printing by a team of mobile robots. Autom. Constr..

[21] Bos F., Wolfs R., Ahmed Z., Salet T. (2018). Large scale testing of digitally fabricated concrete (DFC) elements. Proceedings of the RILEM International Conference on Concrete and Digital Fabrication.

[22] Dedenis M., Sonebi M., Amziane S., Perrot A., Amato G. (2020). Effect of metakaolin, fly ash and polypropylene fibres on fresh and rheological properties of 3d printing based cement materials. Proceedings of the RILEM International Conference on Concrete and Digital Fabrication.

[23] Bester F., Heever M.v.d., Kruger J., Cho S., Zijl G. (2020). van Steel fiber links in 3D printed concrete. Proceedings of the RILEM International Conference on Concrete and Digital Fabrication.

[24] Hameed R., Papon A., Perrot A., Rangeard D. (2020). Effect of Metallic Fibers on the Print Quality and Strength of 3D Printed Concrete. Proceedings of the RILEM International Conference on Concrete and Digital Fabrication.

[25] Arunothayan A.R., Nematollahi B., Ranade R., Bong S.H., Sanjayan J.G., Khayat K.H. (2021). Fiber orientation effects on ultra-high performance concrete formed by 3D printing. Cem. Concr. Res..

[26] Bos F.P., Ahmed Z.Y., Jutinov E.R., Salet T.A. (2017). Experimental exploration of metal cable as reinforcement in 3D printed concrete. Materials.

[27] Luhar S., Suntharalingam T., Navaratnam S., Luhar I., Thamboo J., Poologanathan K., Gatheeshgar P. (2020). Sustainable and renewable bio-based natural fibres and its application for 3D printed concrete: A review. Sustainability.

[28] Caron J.-F., Demont L., Ducoulombier N., Mesnil R. (2021). 3D printing of mortar with continuous fibres: Principle, properties and potential for application. Autom. Constr..

[29] Zollo R.F. (1997). Fiber-reinforced concrete: An overview after 30 years of development. Cem. Concr. Compos..

[30] Boulekbache B., Hamrat M., Chemrouk M., Amziane S. (2010). Flowability of fibre-reinforced concrete and its effect on the mechanical properties of the material. Constr. Build. Mater..

[31] Martinie L., Rossi P., Roussel N. (2010). Rheology of fiber reinforced cementitious materials: Classification and prediction. Cem. Concr. Res..

[32] Farina I., Fabbrocino F., Carpentieri G., Modano M., Amendola A., Goodall R., Feo L., Fraternali F. (2016). On the reinforcement of cement mortars through 3D printed polymeric and metallic fibers. Compos. Part B Eng..

[33] Hambach M., Rutzen M., Volkmer D., Sanjayan J.G., Nazari A., Nematollahi B. (2019). Chapter 5—Properties of 3D-Printed Fiber-Reinforced Portland Cement Paste. 3D Concrete Printing Technology.

[34] Hambach M., Volkmer D. (2017). Properties of 3D-printed fiber-reinforced Portland cement paste. Cem. Concr. Compos..

[35] Jin Y., Zhou X., Chen M., Zhao Z., Huang Y., Zhao P., Lu L. (2022). High toughness 3D printed white Portland cement-based materials with glass fiber textile. Mater. Lett..

[36] Shakor P., Sanjayan J., Nazari A., Nejadi S. (2017). Modified 3D printed powder to cement-based material and mechanical properties of cement scaffold used in 3D printing. Constr. Build. Mater..

[37] Rutzen M., Schulz M., Moosburger-Will J., Lauff P., Fischer O., Volkmer D. (2021). 3D printing as an automated manufacturing method for a carbon fiber-reinforced cementitious composite with outstanding flexural strength (105 N/mm^2^). Mater. Struct..

[38] Rubio M., Sonebi M., Amziane S. (2017). 3D printing of fibre cement-based materials: Fresh and rheological performances. Acad. J. Civ. Eng..

[39] Li H., Liebscher M., Michel A., Quade A., Foest R., Mechtcherine V. (2021). Oxygen plasma modification of carbon fiber rovings for enhanced interaction toward mineral-based impregnation materials and concrete matrices. Constr. Build. Mater..

[40] Mechtcherine V., Michel A., Liebscher M., Schneider K., Großmann C. (2020). Mineral-impregnated carbon fiber composites as novel reinforcement for concrete construction: Material and automation perspectives. Autom. Constr..

[41] Harbouz I., Yahia A., Roziere E., Loukili A. (2023). Printing quality control of cement-based materials under flow and rest conditions. Cem. Concr. Compos..

[42] Mechtcherine V., Van Tittelboom K., Kazemian A., Kreiger E., Nematollahi B., Nerella V.N., Santhanam M., De Schutter G., Van Zijl G., Lowke D. (2022). A roadmap for quality control of hardening and hardened printed concrete. Cem. Concr. Res..

[43] Nicolas R., Richard B., Nicolas D., Irina I., Temitope K.J., Dirk L., Viktor M., Romain M., Arnaud P., Ursula P. (2022). Assessing the fresh properties of printable cement-based materials: High potential tests for quality control. Cem. Concr. Res..

[44] Roussel N., Coussot P. (2005). “Fifty-cent rheometer” for yield stress measurements: From slump to spreading flow. J. Rheol..

[45] Ducoulombier N., Mesnil R., Carneau P., Demont L., Bessaies-Bey H., Caron J.-F., Roussel N. (2021). The “Slugs-test” for extrusion-based additive manufacturing: Protocol, analysis and practical limits. Cem. Concr. Compos..

[46] Jacquet Y., Picandet V., Rangeard D., Perrot A. (2020). Gravity induced flow to characterize rheological properties of printable cement-based materials. RILEM Tech. Lett..

[47] Perrot A., Rangeard D., Pierre A. (2016). Structural built-up of cement-based materials used for 3D-printing extrusion techniques. Mater. Struct..

[48] Roussel N. (2018). Rheological requirements for printable concretes. Cem. Concr. Res..

[49] Wolfs R.J.M., Bos F.P., Salet T.A.M. (2018). Early age mechanical behaviour of 3D printed concrete: Numerical modelling and experimental testing. Cem. Concr. Res..

[50] Li S., Jensen O.M., Yu Q. (2022). Influence of steel fiber content on the rate-dependent flexural performance of ultra-high performance concrete with coarse aggregates. Constr. Build. Mater..

